# Structure‐Guided Identification and Evaluation of Epalrestat and Ranirestat‐Like Compounds Against Aldose Reductase: Therapeutic Management of Diabetic Neuropathy

**DOI:** 10.1002/open.202500110

**Published:** 2025-08-22

**Authors:** Mohd Shahnawaz Khan, Dharmendra Kumar Yadav, Moyad Shahwan, Anas Shamsi

**Affiliations:** ^1^ Department of Biochemistry College of Science King Saud University Riyadh 11451 Kingdom of Saudi Arabia; ^2^ Gachon Institute of Pharmaceutical Science and Department of Pharmacy College of Pharmacy Gachon University Incheon 21936 Republic of Korea; ^3^ Department of Clinical Sciences, College of Pharmacy and Health Sciences Ajman University Ajman UAE; ^4^ Center of Excellence in Precision Medicine and Digital Health Department of Physiology, Faculty of Dentistry Chulalongkorn University Bangok Thailand; ^5^ Centre of Medical and Bio‐Allied Health Sciences Research Ajman University Ajman 346 UAE

**Keywords:** aldose reductase inhibitors, binding affinity, diabetic neuropathy, molecular dynamics simulations, virtual screening

## Abstract

Aldose reductase (ALDR) is a critical protein involved in the pathogenesis of diabetic complications such as retinopathy, neuropathy, and nephropathy. Due to the activation of inflammatory and cytotoxic pathways under hyperglycemic conditions, ALDR has become an important target for therapeutic development. Currently, available drugs such as epalrestat and ranirestat are suboptimal due to factors such as toxicity and low solubility. In this study, a structure‐based approach was used to screen the PubChem database to identify novel ALDR inhibitors with a Tanimoto coefficient greater than 0.8 with the structural frameworks of epalrestat and ranirestat. A systematic virtual screening, including molecular docking, drug‐likeness assessment, and molecular dynamics (MD) simulations, revealed two promising candidates, PubChem CIDs: 45110135 and 58643777. These compounds showed higher binding and selectivity toward ALDR than epalrestat and ranirestat in docking studies. MD simulations supported the stability and preferred dynamics of their interactions with ALDR. These findings suggest that compounds CID:45110135 (N‐[3‐fluoro‐4‐(4‐fluoro‐1,3‐dioxoisoindol‐2‐yl)phenyl]pyridine‐2‐carboxamide) and CID:58643777 ([(5Z)‐4‐oxo‐2‐sulfanylidene‐5‐[[3‐[3‐(trifluoromethyl)phenyl]phenyl]methylidene]−1,3‐thiazolidin‐3‐yl]propanoic acid) might have the potential to be lead compounds for the development of new drugs for diabetic neuropathy after required validation.

## Graphical abstract

Structure‐based virtual screening of PubChem compounds identifies two novel epalrestat and ranirestat‐like inhibitors with improved affinity and pharmacokinetic profiles targeting aldose reductase. Molecular docking, ADMET filtering, and 500‐ns molecular dynamics simulations confirm the stability and interaction specificity of the candidates, highlighting their potential for diabetic neuropathy therapy.

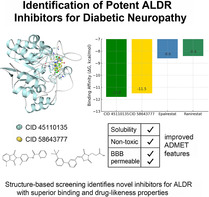

## 1 Introduction

Diabetic peripheral neuropathy is one of the most common microvascular complications of diabetes mellitus, occurring in 60%–70% of patients in the USA.^[^ ^1^ ^]^ In Europe, particularly in countries like the UK, prevalence rates range from 25% to 30%, with the EURODIAB study reporting 28% among type 1 diabetics.^[^ ^2^ ^]^ In Asia, diabetic peripheral neuropathy prevalence varies widely by country, from 17% to 62% in China, 9.6% to 78% in India (29% in North India vs. 47% in South India), and around 33.3% in Gulf nations like Saudi Arabia.^[^ ^2^ ^,^ ^3^ ^]^ These figures underscore the global burden of diabetic peripheral neuropathy and the need for routine screening and early management across diverse populations. It is a significant cause of increased morbidity and mortality in diabetic patients, resulting in more hospitalizations than all other diabetic complications and over 60% of nontraumatic amputations.^[^ ^4^ ^]^ This heterogeneous condition affects the sensory, motor, and autonomic nerves of the peripheral nervous system and may manifest through a wide range of clinical and subclinical symptoms.^[^ ^5^ ^]^ Sensory nerve damage manifests as pain, numbness, and tingling, while motor nerve impairment leads to muscle weakness and gait abnormalities. Autonomic neuropathy causes cardiovascular instability (e.g., orthostatic hypotension) and gastrointestinal dysfunction. These complications markedly reduce quality of life and elevate mortality risk. However, the development of diabetic neuropathy is different in type 1 and type 2 diabetes.^[^ ^6^ ^]^ Diabetic neuropathy occurs early in type 2 diabetes but may take 1–25 years to develop in patients with type 1 diabetes.^[^ ^7^ ^]^ Aldose reductase (ALDR), an enzyme of the polyol pathway, is widely recognized as the primary mediator of diabetic neuropathy.^[^ ^8^ ^]^ In a high glucose environment, ALDR transforms glucose into sorbitol that builds up within cells and causes osmotic stress, oxidative stress, and cell dysfunction.^[^ ^9^ ^]^ This mechanism is responsible for nerve damage and other related complications, such as diabetic retinopathy and nephropathy.^[^ ^10^ ^]^ Thus, ALDR inhibitors (ALDRIs) that block ALDR have demonstrated the possibility of reversing these pathological consequences and may be used as a treatment for diabetic complications.^[^ ^8^ ^]^

Two of the clinically tested ALDRIs are epalrestat and ranirestat, which have been shown to have these effects on sorbitol levels and the symptoms of diabetic neuropathy.^[^ ^11^ ^]^ epalrestat, which is available in Japan, has shown the advantages of the drug in controlling neuropathic pain, enhancing vibration sensation, and decreasing the rate of heart irregularities.^[^ ^12^ ^]^ However, these drugs have numerous problems, such as low solubility and side effects that hinder their use in various applications.^[^ ^13^ ^]^ Although clinical trials have yielded variable results, increasing data support an essential function of ALDR in diabetic neuropathy, further emphasizing the need for improved ALDRIs.^[^ ^14^ ^]^ ALDRIs like Fidarestat and Zenarestat show improved solubility but face challenges such as hepatotoxicity and short half‐lives.^[^ ^15^ ^]^ MK204, a spiroindoline derivative, exhibits sub‐nanomolar IC50 but poor oral bioavailability.^[^ ^16^ ^]^ New developments in computational biology and structure‐based drug design are promising ways to find and improve ALDRIs.^[^ ^17^ ^]^ Using virtual screening and molecular modeling approaches, the researchers can rank large compound databases and select the best candidates with high binding affinities and pharmacokinetic profiles.^[^ ^18^ ^]^ This work used an integrative computational method to search for other ALDRIs that share structural similarities to epalrestat and ranirestat. To identify compounds with high structural similarity to the screened compounds, compounds with an 80% or higher Tanimoto similarity score were selected from PubChem with regard to binding affinities and drug‐likeness.

For the top screened candidates, ADMET profiling and biological activity prediction by PASS analysis^[^ ^19^ ^]^ were performed. The stability and dynamic properties of the ALDR‐compound complexes were investigated using all‐atom molecular dynamics (MD) simulations. Analysis of the structural quality was done using root mean square deviation (RMSD), root mean square fluctuation (RMSF), radius of gyration (*R*g), solvent accessible surface area (SASA), free energy landscapes (FEL), hydrogen bond dynamics, and principal component analysis (PCA). Among the evaluated compounds, CID:45110135 and CID:58643777 showed higher stability, selectivity, and binding affinity than epalrestat and ranirestat and can be considered promising for further work. This work also highlights the potential of structure‐guided approaches in drug discovery. It could be used to guide the design of new ALDRIs for diabetic neuropathy that may overcome the current drawbacks of the treatment and offer better outcomes for patients.

## 2 Results and Discussion

### 2.1 Epalrestat and Ranirestat‐Like Compounds and Virtual Screening

A PubChem database search yielded 1,815 compounds with a Tanimoto coefficient of at least 0.8, relative to epalrestat and ranirestat. The structures of these compounds were first assessed to determine their binding affinities and orientations with ALDR. Some of the screened molecules were found to have very good binding affinities, which qualified them as potential ALDRIs. The docking study showed that the top 10 compounds had affinity scores higher than −11.5 kcal mol^–^^1^ with ALDR and greater than the values of epalrestat and ranirestat (**Table** **1**). The pharmacophore of ALDRIs typically includes a planar aromatic system and a polar group. Our candidates, CID:45110135 and CID:58643777, retain these features while introducing novel moieties (e.g., trifluoromethyl groups) to enhance binding (Table 1). Therefore, the identified compounds with the best binding affinities were considered worthy of further investigation as potential selective ALDRIs that could be of relevance to managing diabetic neuropathy.

**Table 1:** List of selected compounds and their docking scores with ALDR.

S. No.	Compound CID	Chemical Structure	Binding Affinity [kcal mol^−^^1^]	p*K*i	Ligand Efficiency [kcal/mol/nonH atom]	Torsional Energy
1.	122647443	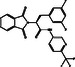	−12.7	9.31	0.3735	1.8678
2.	155539848	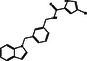	−12.6	9.24	0.4846	1.5565
3.	69200154	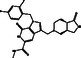	−12.4	9.09	0.3647	1.5565
4.	16114380	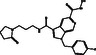	−12.3	9.02	0.3727	2.8017
5.	60117795	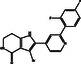	−12.2	8.95	0.488	0.6226
6.	134793525	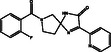	−12.0	8.8	0.48	0.6226
7.	60118694	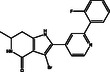	−12.0	8.8	0.48	0.6226
8.	89792047	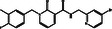	−12.0	8.8	0.4444	1.5565
9.	45110135	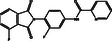	−11.8	8.65	0.4214	0.9339
10.	58643777	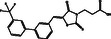	−11.5	8.43	0.3966	2.1791
11.	Epalrestat	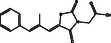	−8.6	6.31	0.4095	1.5565
12.	Ranirestat	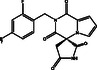	−8.4	6.16	0.3231	0.6226

### 2.2 Hit Selection and Drugability Assessment

The pharmacokinetic properties of the screened compounds were determined using pkCSM in order to get valuable information about their molecular properties and their likelihood of being a drug. ADMET properties were predicted to analyze the bioactivity, pharmacokinetics, and toxicity of the compounds (**Table** **2**). Among the screened compounds, two were identified as promising candidates: Ranirestat‐like compound (PubChem CID: 45110135) and epalrestat‐like compound (PubChem CID: 58643777) because of better ADMET profiles without any toxicity. In carcinogenicity predictions, both compounds were identified as noncarcinogenic, which is evidence of their possible therapeutic nature. The ADMET profiles of CID:45110135 and CID:58643777 highlight their advantages over existing ALDRIs, particularly in terms of safety and metabolic stability. Their lack of hERG inhibition, noncarcinogenic nature, and favorable excretion properties position them as superior candidates for further development. These results suggest that they are well‐suited for more complex preclinical and clinical research.

**Table 2:** ADMET properties of the screened molecules. Where HIA, human intestinal absorption; BBB, blood‐brain barrier; OCT2, organic cation transporter 2; hERG, human ether‐a‐go–go‐related gene.

Compound CID	Absorption		Distribution	Metabolism	Excretion	Toxicity		
	HIA	Oral Bioavailability	BBB Permeability	CYP2C19 inhibitor	Renal OCT2 Inhibitor	hERG Blockers	NR‐AhR	SR‐ARE
122647443	Yes	Yes	Penetrable	Yes	No	Toxic	Safe	Toxic
155539848	Yes	Yes	Penetrable	Yes	No	Toxic	Toxic	Toxic
69200154	Yes	Yes	Penetrable	No	No	Safe	Safe	Toxic
16114380	Yes	Yes	Penetrable	Yes	No	Safe	Safe	Toxic
60117795	Yes	Yes	Penetrable	No	No	Toxic	Toxic	Toxic
134793525	Yes	Yes	Penetrable	No	No	Toxic	Safe	Safe
60118694	Yes	Yes	Penetrable	Yes	No	Safe	Toxic	Toxic
89792047	Yes	Yes	Penetrable	Yes	No	Toxic	Toxic	Safe
45110135	Yes	Yes	Penetrable	No	No	Safe	Safe	Safe
58643777	Yes	Yes	Penetrable	No	No	Safe	Safe	Safe
Epalrestat	Yes	Yes	Penetrable	No	No	Safe	Safe	Toxic
Ranirestat	Yes	Yes	Penetrable	No	No	Safe	Safe	Safe

### 2.3 PASS Analysis

PASS analysis was conducted for two selected compounds to evaluate the biological activity and efficacy of the compounds identified through ADMET analysis. **Table** **3** highlights the biological properties of interest, focusing on antidiabetic and antineuropathic activities, along with their respective activity scores (Pa values). The results indicate that compounds CID:45110135 and CID:58643777 exhibit moderate to high potential for these activities, with Pa values ranging from 0.305 to 0.826. The PASS predictions for the reference inhibitors epalrestat and ranirestat confirmed their antidiabetic, antineuropathic, and ALDR inhibitory activities, validating the reliability of the projections. Collectively, these findings suggest that CID:45110135 and CID:58643777 hold promise for the treatment of diabetic neuropathy.

**Table 3:** PASS parameters of the selected hit molecules.

S. No.	Compound	Pa	Pi	Activity
1.	CID:45110135	0,411	0,033	Diabetic neuropathy treatment
		0,391	0,033	Neuropeptide Y2 antagonist
		0,377	0,083	Neurodegenerative diseases treatment
		0,305	0,017	Pancreatic disorders treatment
		0,403	0,213	Nootropic
2.	CID:58643777	0,826	0,003	Insulysin inhibitor
		0,702	0,004	Amyloid beta precursor protein antagonist
		0,637	0,002	ALDR inhibitor
		0,635	0,062	Nootropic
		0,565	0,015	Antidiabetic
3.	Epalrestat	0,788	0,004	Insulysin inhibitor
		0,773	0,004	Antidiabetic symptomatic
		0,432	0,003	Aldose reductase inhibitor
		0,407	0,042	Antidiabetic
		0,292	0,099	Antinephritic
4.	Ranirestat	0,842	0,001	ALDR inhibitor
		0,683	0,004	Antidiabetic symptomatic
		0,300	0,143	Dementia treatment

### 2.4 Interaction Analysis

**Figure 1:**
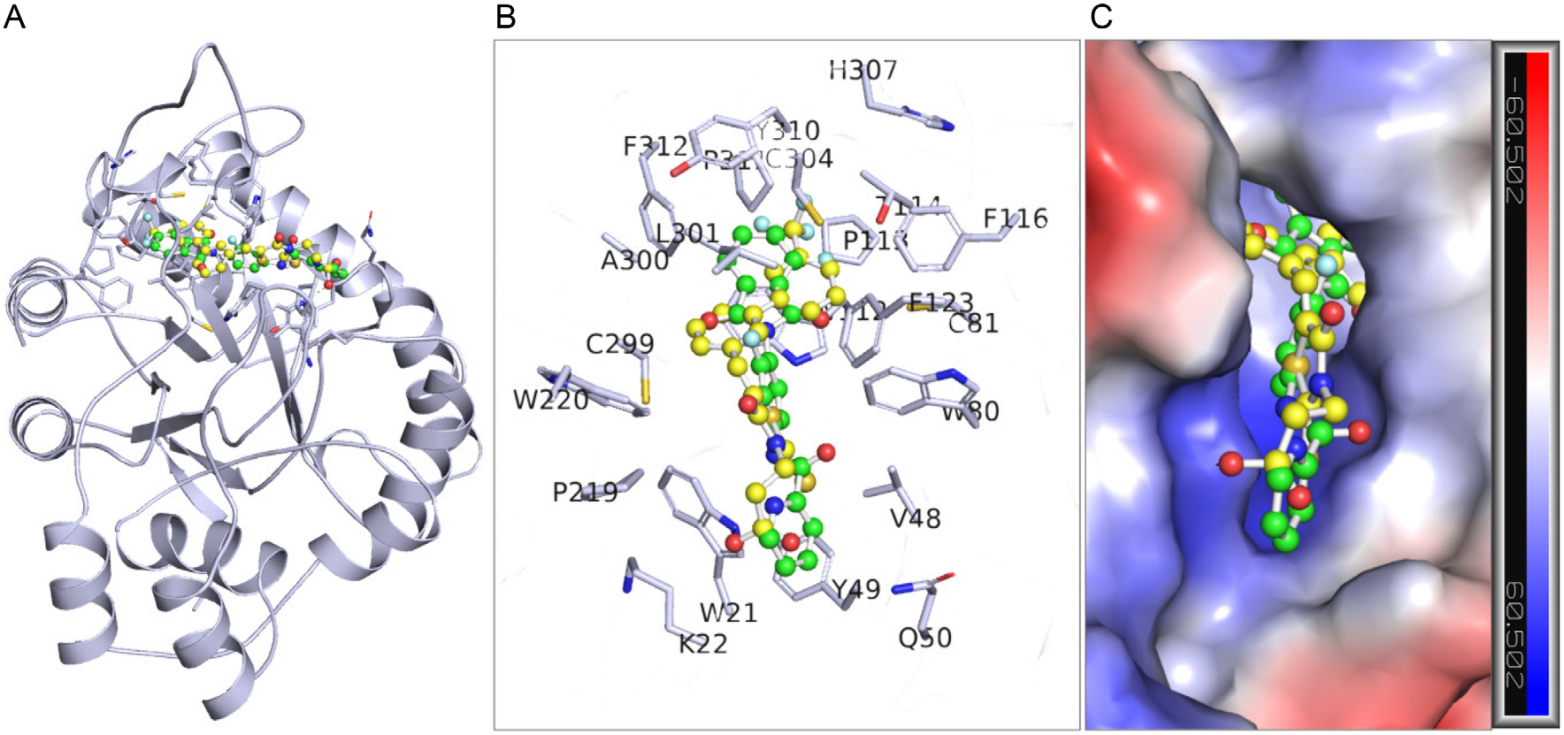
Binding mode of ligand CID:45110135 and CID:58643777 to ALDR. A) Cartoon representation of ALDR complexed with CID:45110135 and CID:58643777. B) Magnified cartoon representation of ALDR interacting residues with CID:45110135 and CID:58643777. C) Surface representation of ALDR complexed with the ligands. CID:45110135 and CID:58643777 are shown in ball‐and‐stick models in green and yellow elements, respectively.

To analyze the interactions between the identified compounds and ALDR, all potential docked conformations of the docking output files of CID:45110135 and CID:58643777 were analyzed. After the molecular docking, ADMET evaluation, and PASS prediction, the interaction of these compounds with ALDR was further studied in detail (**Figure** **1**). This well–developed interaction profile indicates high binding specificity within the ALDR binding site (Figure 1A). CID:45110135 showed multiple conformations with interaction at the active site, especially with Tyr49. At the same time, CID:58643777 also interacted with Tyr49 and had similar interactions as CID:45110135 (Figure 1B). Interaction analysis further revealed that CID:45110135 forms several hydrogen bonds with critical ALDR residues, enhancing binding specificity and contributing to the complex's stability. The binding patterns of CID:45110135 and CID:58643777 show that both compounds are bound inside the deep binding cavity of ALDR (Figure 1C). Collectively, these findings highlight the CID:45110135 and CID:58643777 binding potential and their promise as strong candidates for ALDR inhibition.

**Figure 2:**
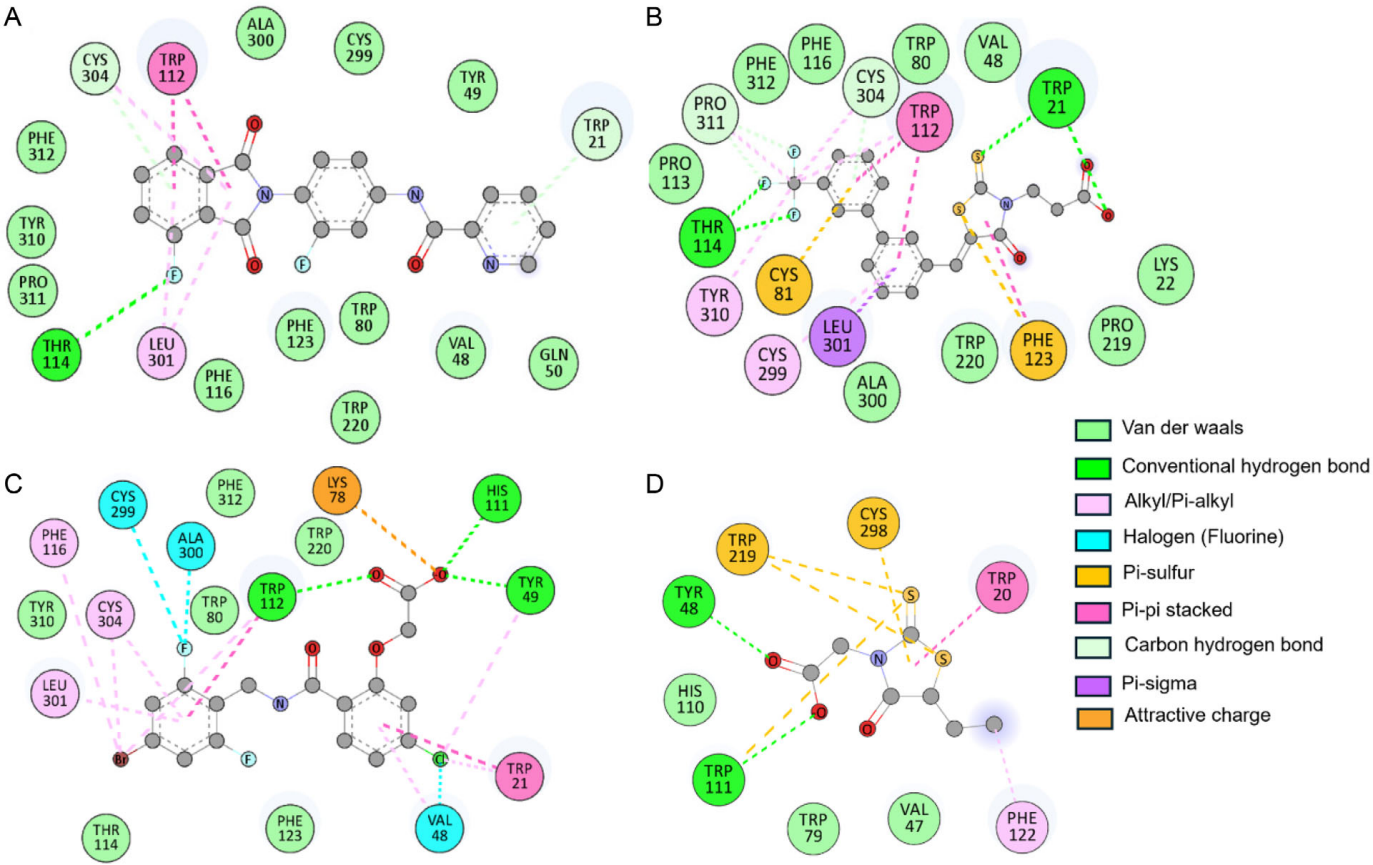
Interactions of CID:45110135 and CID:58643777 to ALDR. A) 2D representation of binding‐pocket residues of ALDR interacting with the ligand CID:45110135, B) CID:58643777, C) MK408 (PDB ID: 4JIR), D) epalrestat (PDB ID: 4JIR).

Both compounds show critical interactions with the active site and its surrounding residues of ALDR. CID:45110135 exhibited interactions including hydrogen bonds with Trp21, Thr114, and Cys304, Pi–Pi stacked with Trp112, alkyl/pi‐alkyl with Leu301, Cys304, and van der Waals with Val48, Tyr49, GIn50, Trp80, Phe116, Phe123, Trp220, Cys299, Ala300, Tyr310, Pro311, and Phe312 (**Figure** **2**A). Similarly, CID:58643777 formed hydrogen bonds with Trp21, Thr114, Cys304, and Pro311, Pi–Pi stacked with Trp112, Phe123, and Alkyl/Pi‐Alkyl with Trp112, Cys299, Cys304, Tyr310, Pro311, and Pi‐sigma with Leu301, Pi‐sulfur with Cys81, Phe123, and van der Waals with Lys22, Val48, Trp80, Pro113, Phe116, Pro219, Trp220, Ala300, and Phe312 interactions (Figure 2B). Notably, 18 interacting residues were shared between both compounds. Compound CID:45110135 was bound at the proton donor active site Tyr49 and NADP^+^ binding site Trp220, while CID:58643777 was bound at the NADP^+^ binding sites Pro219 and Trp220. These interactions showed various common features with reference to ALDR inhibitors, MK408 (PDB ID: 4JIR) and epalrestat (PDB ID: 4JIR) (Figure 2C,D). Trp219 and Trp220 in ALDR are critical for stabilizing the NADP^+^ cofactor and forming *π*‐*π* interactions with inhibitors. CID:45110135 and CID:58643777 exhibited van der Waals interactions with Trp220, mirroring MK408. However, CID:58643777 uniquely engaged Pro219 via van der Waals interaction, a residue not targeted by epalrestat. Both compounds showed stronger *π–*alkyl interactions (e.g., with Leu301 and Cys304) than epalrestat, potentially enhancing binding specificity. Notably, CID:58643777 formed a Pi–sulfur interaction with Cys81, absent in reference inhibitors. The stability and time evolution of these interactions were further studied through 500 ns MD simulations.

### 2.5 Molecular Dynamics Simulations

Molecular dynamic simulations were performed to evaluate the stability and structural changes in ALDR after compound binding.^[^ ^20^ ^]^ GROMACS 2020β^[^ ^21^ ^]^ was utilized to execute a 500 ns simulation. Several parameters, such as RMSD, RMSF, *R*g, and SASA, along with hydrogen bond analysis and PCA, were computed. Initially, we calculate some energies to ensure system stability. The potential energy of ALDR, ALDR‐CID:45110135, and ALDR‐CID:58643777 complexes was −653517, −448925, and −448506 kJ mol^–1^, respectively. Kinetic energy for ALDR, ALDR‐CID:45110135, and ALDR‐CID:58643777 complexes was 130149, 93067.5, and 93010.7 kJ mol^–1^, respectively. The total energy for ALDR, ALDR‐CID:45110135, and ALDR‐CID:58643777 complexes was −523368, −355857, and **−**355495 kJ mol^–1^, respectively. A significant reduction of all three energies was observed after the CID:45110135 and CID:58643777 compounds interacted with ALDR, suggesting the systems’ stability. Various parameters for structural deviation and compactness were studied, as discussed in the ensuing sections.

#### 2.5.1 Overall Structural Deviation and Residual Fluctuation Analysis

**Figure 3:**
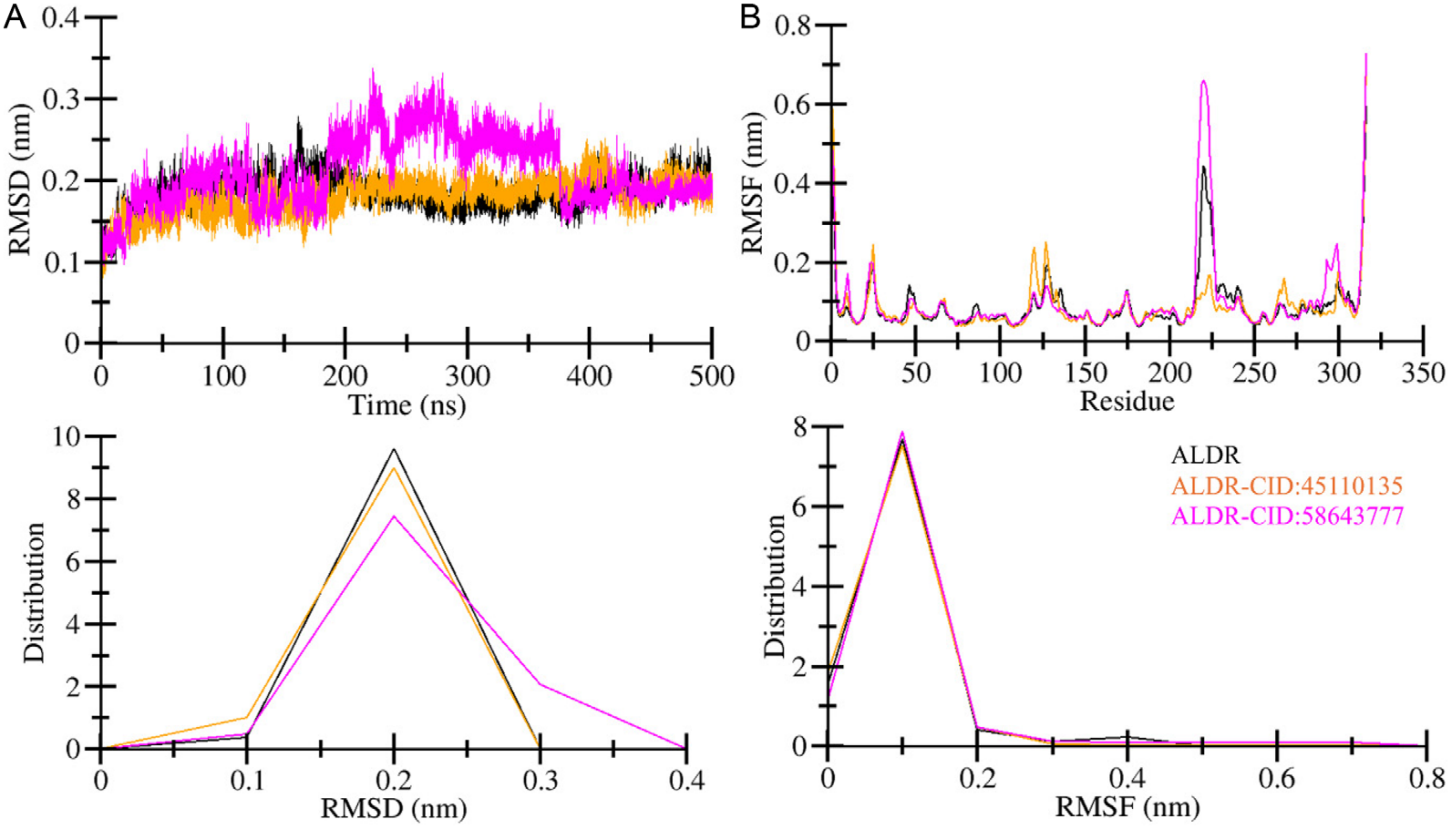
Time‐evolution of structural dynamics of ALDR (black) before and after CID:45110135 (orange) and CID:58643777 (magenta) binding. A) Structural changes within ALDR after compound bindings captured through RMSD analysis. B) Fluctuations of individual residues within ALDR after compound binding. The lower panels represent the distributed data points of RMSD and RMSF values.

The dynamic behavior of ALDR was assessed by comparing the compound binding complexes for 500 ns time. RMSD is a statistical tool widely used to quantify structural deviation in proteins.^[^ ^22^ ^]^ RMSD plot of the protein backbone was computed against time in the ns units (**Figure** **3**A). Average deviation values were calculated for ALDR, ALDR‐CID:45110135, and ALDR‐CID:58,643,777 complexes were 0.18, 0.17, and 0.20 nm, respectively (**Table** **4**). The ALDR‐CID:45110135 complex exhibited a lower RMSD (0.17 nm) than both free ALDR (0.18 nm) and ALDR‐CID:58643777 (0.20 nm), indicating enhanced stability. RMSF values (0.08 nm for ALDR‐CID:45110135 vs. 0.10 nm for ALDR‐CID:58643777) further highlight reduced residual fluctuations in CID:45110135‐bound ALDR. The maximum deviation point was also assessed for ALDR, ALDR‐CID:45110135, and ALDR‐CID:58643777 complexes was 0.27, 0.25, and 0.33 nm, respectively. Calculated values indicate that the ALDR‐CID:58643777 complex shows a bit higher deviation during the simulation. The RMSD plot demonstrates that the ALDR‐CID:45110135 complex (orange) shows a steady pattern throughout the simulation. A minor deviation of around 400 ns was observed for the ALDR‐CID:45110135 complex. While the ALDR‐CID:58643777 complex indicates a comparatively higher deviation between 185  and 375 ns, before and after this deviation, the ALDR‐CID:58643777 complex was converged and equilibrated. At the end of the simulation, the ALDR‐CID:58643777 complex shows stability equilibrated to ALDR. This minor deviation was also demonstrated by the distribution plot, as shown in the PDF in Figure 3A, lower panel. Despite minor deviations, ALDR maintained its overall structural integrity and dynamic behavior upon binding with both CID:45110135 and CID:58643777, suggesting that these compounds are viable candidates for further investigation.

**Table 4:** Various statistical values were calculated for ALDR and its complexes with CID:45110135 and CID:58643777 after 500 ns simulations. Intra H‐bonds, intramolecular hydrogen bonds.

Protein/protein–ligand Complex	RMSD [nm]	RMSF [nm]	*R*g [nm]	SASA [nm^2^]	Intra H‐bonds
ALDR	0.18	0.08	1.93	151.2	221
ALDR‐CID:45110135	0.17	0.08	1.93	150.9	216
ALDR‐CID:58643777	0.20	0.10	1.95	152.7	220

RMSF serves as a metric quantifying the average deviation of individual residues’ position from their mean positions during the specified timeframe and ensemble structures.^[^ ^23^ ^]^ Fluctuations within ALDR at the residual level were computed using the RMSF analysis tool. The RMSF plots of ALDR, ALDR‐CID:45110135, and ALDR‐CID:58643777 complexes were generated using the *gmx rmsf* module (Figure 3B). The average fluctuation value of ALDR, ALDR‐CID:45,110,135, and ALDR‐CID:58643777 complexes was 0.08, 0.08, and 0.10 nm, respectively (Table 4). The RMSF value of the ALDR‐CID:58643777 complex shows minor fluctuation during simulation. As the RMSF plot shows, no major fluctuation was observed except for minor fluctuations of the ALDR‐CID:58643777 complex residues between 217 and 223 positions. The distribution plot as PDF also shows a similar pattern of RMSF for ALDR, ALDR‐CID:45110135, and ALDR‐CID:58643777 complexes (Figure 3B, lower panel). Overall, the RMSF analysis highlights the structural stability of ALDR and its complexes.

#### 2.5.2 Structural Compactness and Folding Mechanism Prediction

**Figure 4:**
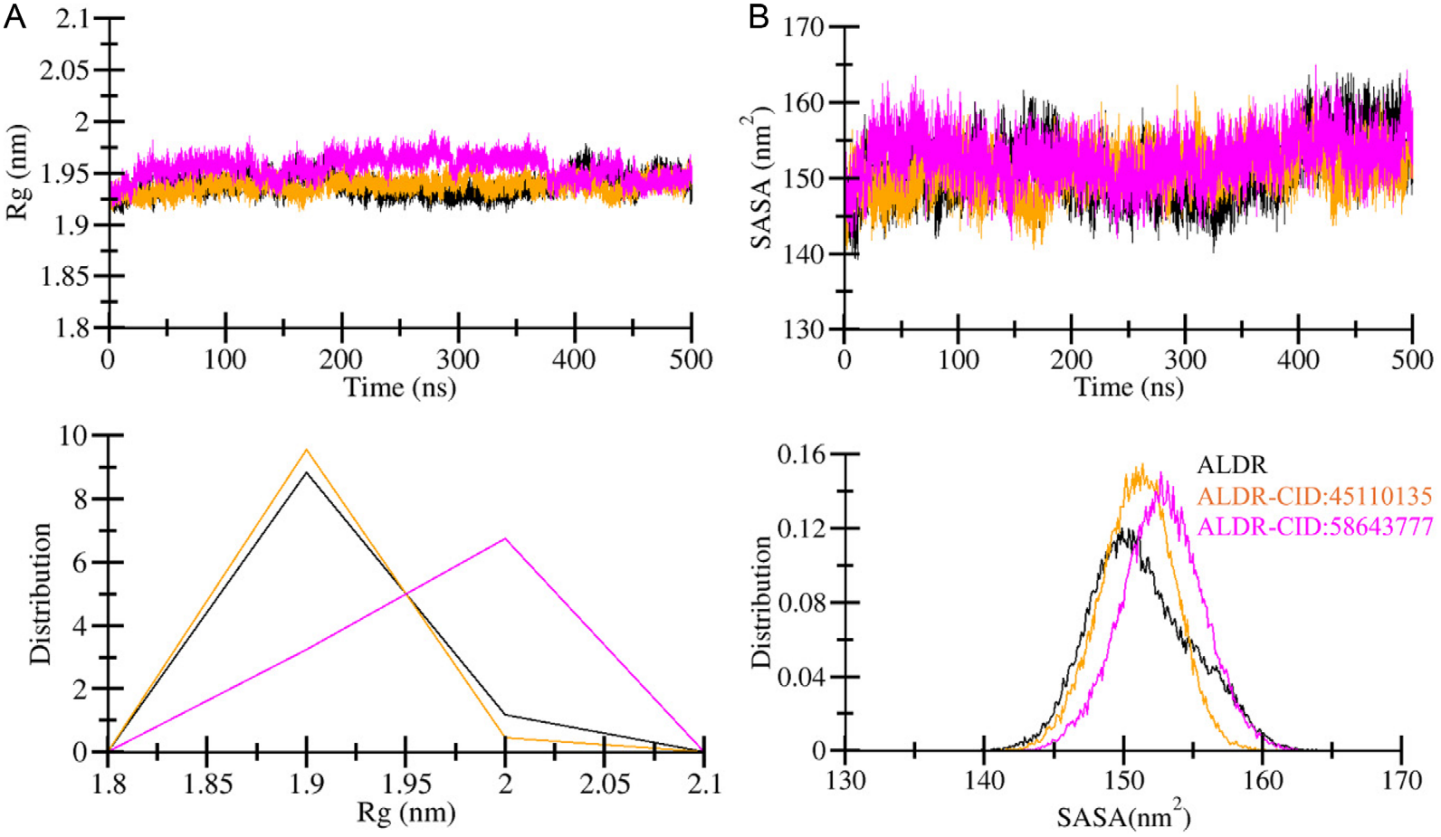
Time‐evolution of structural compactness of ALDR (black) before and after CID:45110135 (orange) and CID:58643777 (magenta) binding**.** A) Structural compactness of ALDR after molecule binding captured through *R*g analysis. B) The folding mechanism of ALDR after molecule binding was monitored through SASA analysis. The lower panels represent the distributed data points of RMSD and RMSF values.

The *R*g was utilized to assess the compactness of ALDR over compound binding. The calculated *R*g for ALDR, ALDR‐CID:45110135, and ALDR‐CID:58643777 complexes are displayed in **Figure** **4**A. The average *R*g values of ALDR, ALDR‐CID:45110135, and ALDR‐CID:58643777 complexes were 1.93, 1.93, and 1.95 nm, respectively (Table 4). Maximum *R*g values for ALDR, ALDR‐CID:45110135, and ALDR‐CID:58643777 complexes were 1.97, 1.97, and 1.99 nm, respectively. ALDR and ALDR‐CID:45110135 show similar stability, while a minor increment in the ALDR‐CID:58643777 complex gyration value was observed. As the *R*g plot indicates, the ALDR‐CID:58643777 complex shows a slightly higher deviation from 150 to 375 ns; later, the complex achieved a similar pattern, which shows stability. The PDF analysis also confirmed the variation in *R*g values of ALDR, ALDR‐CID:45110135, and ALDR‐CID:58643777 complexes (Figure 4A, lower panel).

Solvent‐accessible surface area refers to a critical part of the target protein area that can be occupied by solvent during the simulations.^[^ ^24^ ^]^ SASA was computed using the *gmx sasa* module to understand the conformational behavior of ALDR, ALDR‐CID:45110135, and ALDR‐CID:58643777 complexes. Increasing the order of SASA during the simulation suggests an unfolding mechanism of the protein. In contrast, decreasing the order of the SASA value recommends a higher compactness of the system. The computed SASA plot of ALDR, ALDR‐CID:45110135, and ALDR‐CID:58643777 complexes are displayed in Figure 4B, and the average values are given in Table 4. The calculated average SASA values for ALDR, ALDR‐CID:45110135, and ALDR‐CID:58643777 complexes were 151.2, 150.9, and 152.7 nm^2^, respectively. Maximum SASA reaching points for ALDR, ALDR‐CID:45110135, and ALDR‐CID:58643777 complexes were 163.9, 162.3, and 164.9 nm^2^, respectively. After initial adjustment of the complexes, converged and stable behavior was seen throughout the simulation, as indicated by SASA plots. The PDF plot also shows minimal variation in SASA distribution values (Figure 4B, lower panel). In summary, the Rg and SASA analyses collectively demonstrate that ALDR maintains structural integrity and stability upon binding with both CID:45110135 and CID:58643777, with only minor variations in compactness and solvent exposure.

#### 2.5.3 Hydrogen Bonds Analysis

**Figure 5:**
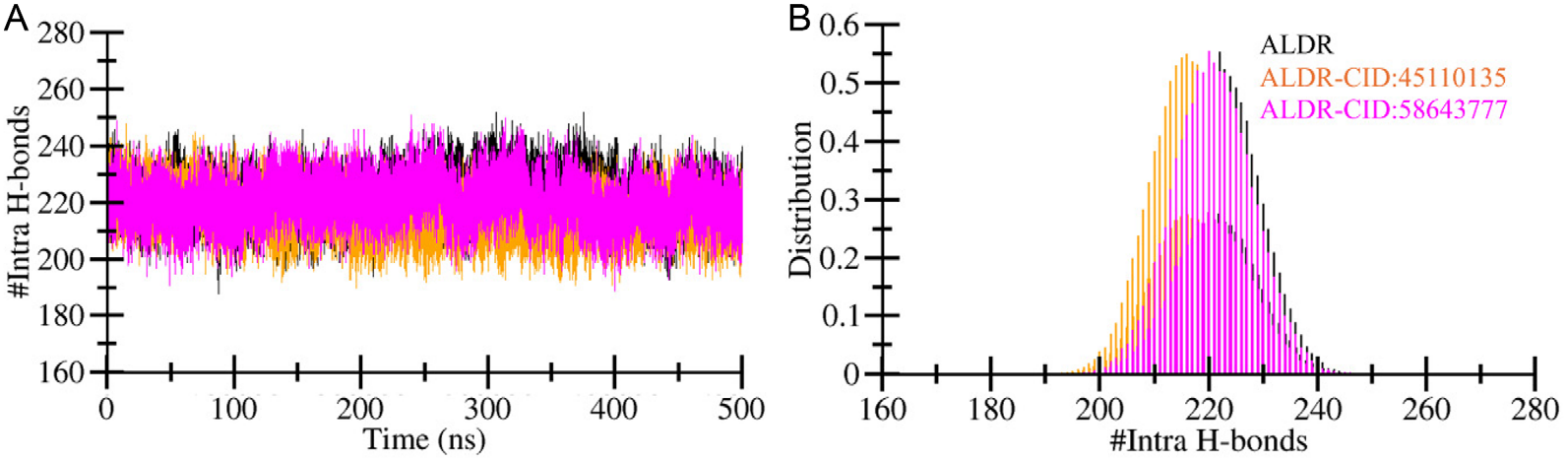
Dynamics of intramolecular hydrogen bonds. A) Changes in intramolecular hydrogen bonds within the ALDR structure were monitored after CID:45110135 and CID:58643777 binding. B) The probability distribution function plot showed the distributed data points of intramolecular hydrogen bond values.

Hydrogen bonds play a critical role in maintaining the structure, stability, and function of proteins.^[^ ^25^ ^]^ Intramolecular hydrogen bonds are critical bonds for structural stability, dynamic behavior, and function of the protein.^[^ ^26^ ^]^ Intramolecular hydrogen bonds are formed within a single molecule between different atoms or groups. The computed plot of intramolecular hydrogen bonds for ALDR, ALDR‐CID:45110135, and ALDR‐CID:58643777 complexes are displayed in **Figure** **5**. Average intramolecular hydrogen bonds for ALDR, ALDR‐CID:45110135, and ALDR‐CID:58643777 complexes were 221, 216, and 220, respectively. Maximum intramolecular hydrogen bonds for ALDR, ALDR‐CID:45110135, and ALDR‐CID:58643777 complexes were 253, 248, and 254, respectively (Figure 5A). The ALDR‐CID:58643777 complex shows one new bond, while the reduction in hydrogen bonds for ALDR‐CID:45110135 was found to be decreased. Despite these minor variations, the observed changes in intramolecular hydrogen bonding were insufficient to induce significant structural perturbations or unfolding. This indicates that the overall structural stability of ALDR remains preserved following compound binding. This observation highlights the resilience of the protein's hydrogen‐bonding network, even upon ligand interaction. The PDF analysis also reveals a similar trend of intramolecular hydrogen bonds for ALDR, ALDR‐CID:45110135, and ALDR‐CID:58643777 complexes (Figure 5B). The similar patterns in the PDF analysis support the conclusion that the binding of these compounds does not disrupt the inherent stability of ALDR.

**Figure 6:**
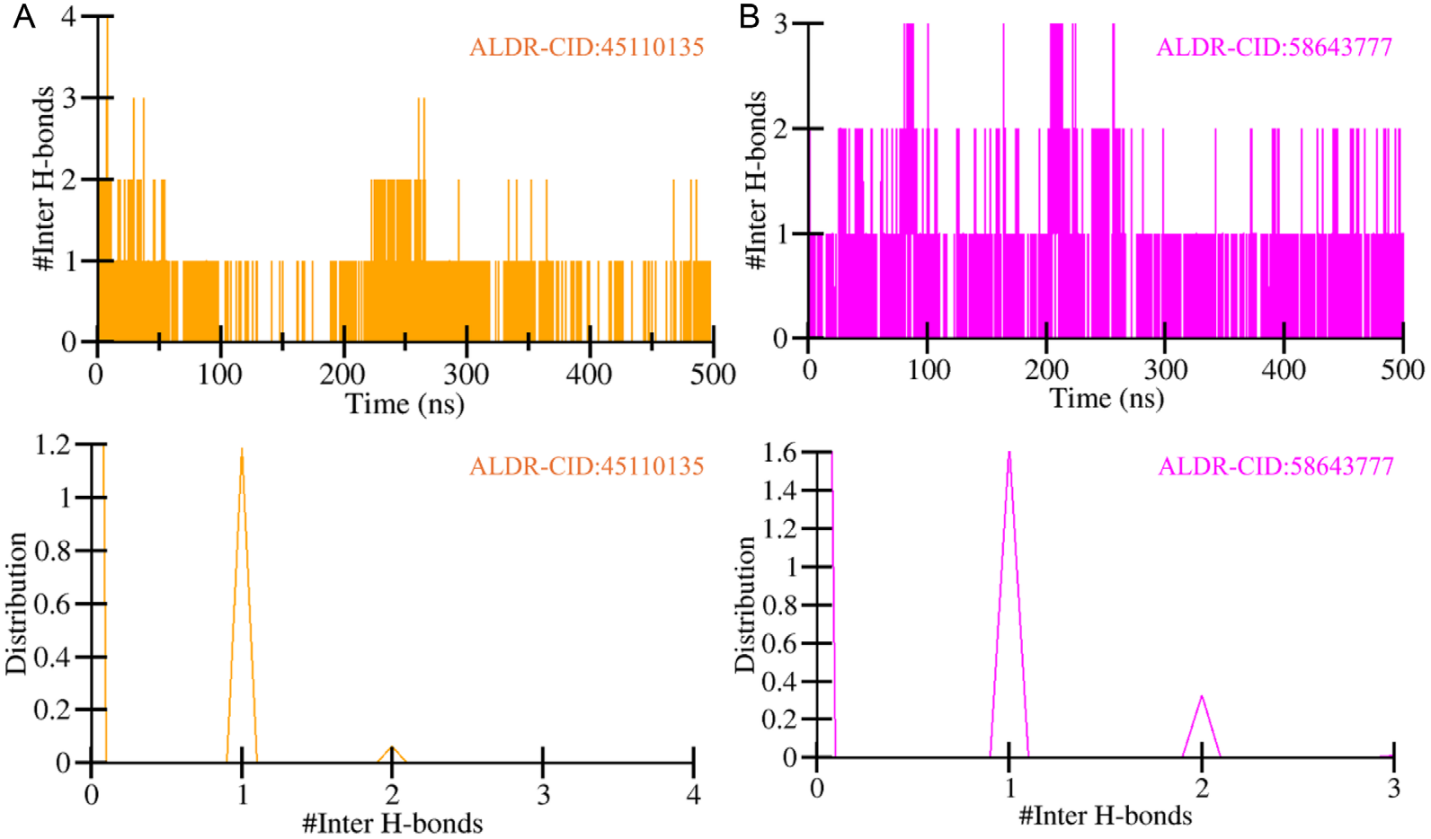
Dynamics of intermolecular hydrogen bonds. A) Representation of intermolecular hydrogen bonds between the ALDR‐CID:45110135 complex. B) Representation of intermolecular hydrogen bonds between the ALDR‐CID:58643777 complex. The lower panels represent the distributed data points of RMSD and RMSF values.

Furthermore, intermolecular hydrogen bonds were analyzed to assess the stability and strength of the protein‐ligand interactions within the ALDR‐CID:45110135 and ALDR‐CID:58643777 complexes. These bonds are critical for maintaining the structural integrity of the complexes during dynamic simulations.^[^ ^27^ ^]^ The calculated intermolecular hydrogen bonds for ALDR‐CID:45110135 and ALDR‐CID:58643777 complexes were 4 and 3, respectively (**Figure** **6**). The analysis revealed that both complexes consistently exhibited at least one intermolecular hydrogen bond throughout the simulation (Figure 6A,B). These bonds demonstrated higher distribution patterns and remained stable over the simulation timeframe (Figure 6, lower panels). The persistent nature of these intermolecular hydrogen bonds highlights the stability of the protein‐ligand complexes. Such interactions are pivotal for ensuring ligand orientation retention and fostering effective binding dynamics. These findings reinforce the structural robustness of the ALDR complexes with CID:45110135 and CID:58643777, suggesting their potential as reliable candidates for further investigation in drug design studies.

#### 2.5.4 Secondary Structure Analysis

**Figure 7:**
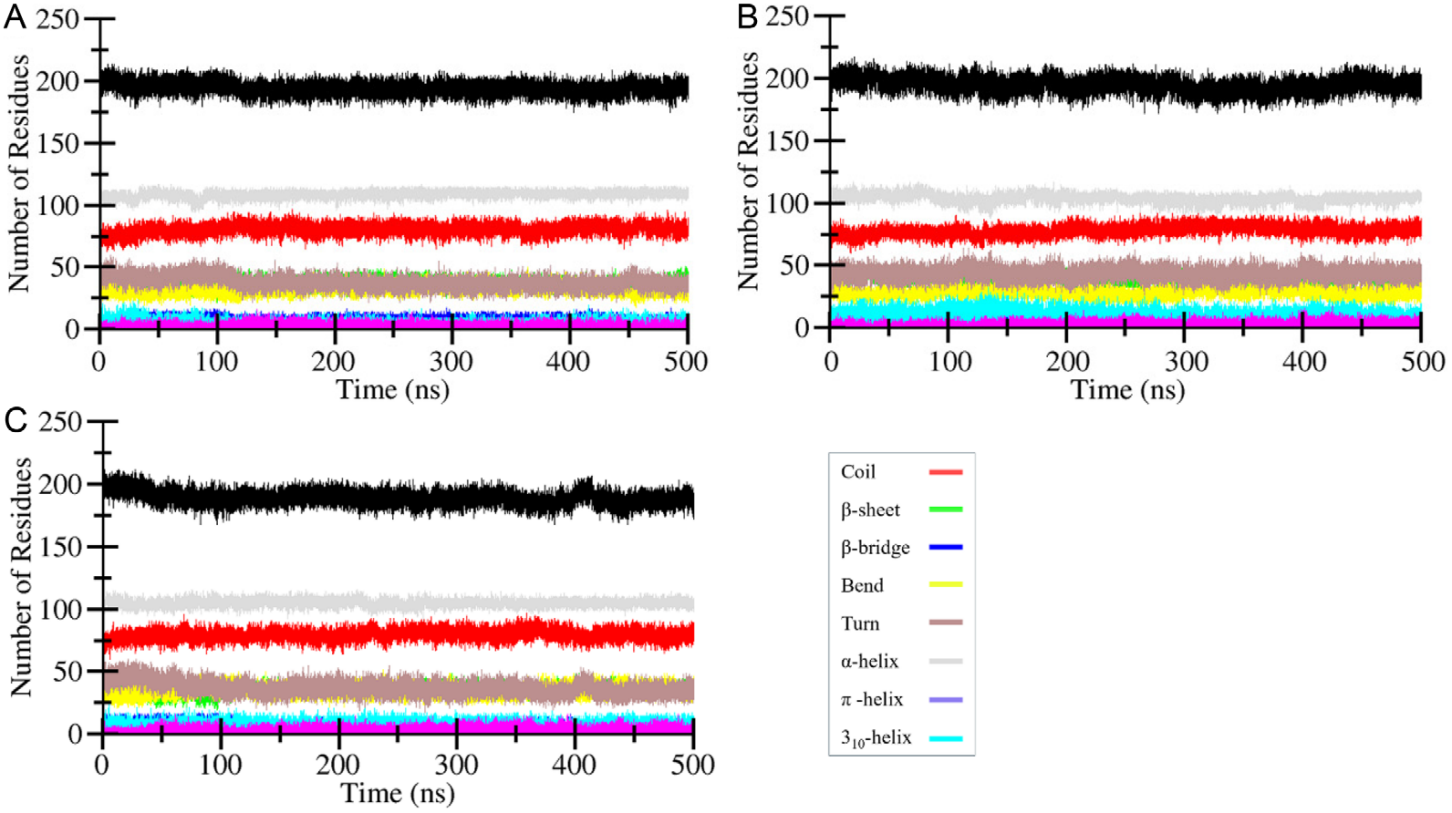
Involvement of residues predicted during secondary structure analysis of A) ALDR, B) ALDR‐CID:45110135, and C) ALDR‐CID:58643777 complex.

Secondary structure elements in ALDR were computed to capture changes that occurred during the simulation over compound binding. DSSP program,^[^ ^28^ ^]^ along with the GROMACS module, was utilized for the analysis of the 500 ns simulation trajectory. The secondary structure plots of ALDR, ALDR‐CID:45110135, and ALDR‐CID:58643777 complexes are presented in **Figure** **7**. Different colors indicate associated secondary structure elements such as *α*‐helices, β‐sheets, β‐bridges, and turns. Changes that occurred during the simulation were calculated and presented in **Table** **5**. Residual involvement was increased after CID:45110135 compound binding during β‐sheet, turn, and 3_10_‐helix formation, and PPII‐helix was consistent after CID:58643777 compound binding residual involvement during β‐bridge, bend, and 3_10_‐helix formation (Figure 7A). Minor decrement was also observed during coil and *α*‐helix formation after CID:45110135 and CID:58643777 compound bindings (Figure 7B,C). The analysis revealed no major changes, which suggests that ALDR was stable during the simulation.

**Table 5:** Secondary structure elements of ALDR computed by 500 ns MD simulation trajectories.

Complex	Structure	Coil	β‐Sheet	β‐Bridge	Bend	Turn	α‐Helix	Pi‐Helix	3_10_‐Helix	PPII‐Helix
ALDR	0.62	0.26	0.12	0.03	0.10	0.12	0.34	0.00	0.02	0.01
ALDR‐CID:45110135	0.62	0.25	0.13	0.02	0.09	0.14	0.33	0.00	0.03	0.01
ALDR‐CID:58643777	0.60	0.25	0.12	0.03	0.11	0.12	0.33	0.00	0.03	0.01

### 2.6 Principal Component Analysis

**Figure 8:**
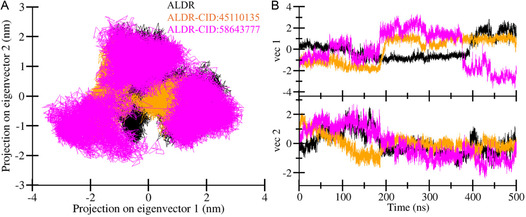
PCA plots showing conformational landscapes of ALDR. A) 2D representation of PCA plot showing conformational landscapes of ALDR before and after compound binding. B) Time‐based eigenvector plot with two initial principal components from PCA.

PCA is a powerful statistical method widely used in MD simulations to isolate and interpret the collective motions of biomolecules.^[^ ^29^ ^]^ The PCA plot of the first two principal components generated for ALDR, ALDR‐CID:45110135, and ALDR‐CID:58643777 complexes are given in **Figure** **8**. The plot shows that both ALDR‐CID:45110135 and ALDR‐CID:58643777 complexes overlapped with ALDR, showing similar conformational states with some wider emotional space occupancy by the ALDR‐CID:58643777 complex (Figure 8A). The vibrational space occupied by ALDR at PC1 was −1.5 to 2.3 nm, and at PC2, −1.7 to 2.5 nm. The space covered by ALDR‐CID:45110135 complexes at PC1 −2.2 to 1.8 nm and at PC2 −1.6 to 2.1 nm. The space covered by the ALDR‐CID:58643777 complex was at PC1−3.6 to 3.2 nm and at PC2 −2.3 to 2.6 nm. The findings show that the ALDR‐CID:58643777 complex achieved a distinguished stable conformational state due to binding adjustment during the simulation. Moreover, a time‐dependent eigenvector plot for the first two principal components was also generated, which is presented in Figure 8B. At first, a vector between 200 and 400 ns time complex deviation was observed, while at vector 2, no major deviation was seen throughout the simulation. PCA and eigenvector analysis suggested that both ALDR‐CID:45110135 and ALDR‐CID:58643777 complexes were stable during the 500 ns simulation.

### 2.7 Free Energy Landscape Analysis

**Figure 9:**
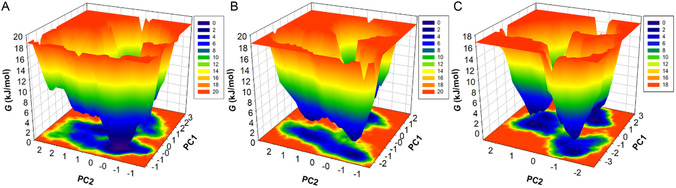
3D contour maps of FEL for A) ALDR, B) ALDR‐CID:45110135, and C) ALDR‐CID:58643777 complex.

Gibbs's free energy landscape is a widely utilized technique that provides insight into the folding mechanism of a protein structure.^[^ ^30^ ^]^ We constructed a FELs plot to understand conformational changes during the unfolding of ALDR over the binding of CID:45110135 and CID:58643777 complexes (**Figure** **9**). The 3D FELs were constructed to describe energy flow from the high‐temperature unfolded state to the low‐energy level folded state of the protein. The ALDR contour map had a single dark blue basin confined within 2–3 native conformational states (Figure 9A). In contrast, the ALDR‐CID:45110135 complex had two different large blue basins associated with two distinct conformational states (Figure 9B). At the same time, the ALDR‐CID:58643777 complex map shows three distinguished low‐energy basins and three different energy funnels related to the other folded states (Figure 9C). The energy funnels observed for both ALDR‐CID:45110135 and ALDR‐CID:58643777 complexes demonstrate their ability to quickly transition between stable conformations, suggesting a degree of structural adaptability. The findings highlight that ALDR achieves stable conformational states following binding with CID:45110135 and CID:58643777. These results underscore the role of ligand binding in modulating the conformational flexibility and energy landscape of the protein, which is critical for understanding protein function and stability in a dynamic environment.

## 3 Conclusions

ALDR has become a promising target for the development of antidiabetic neuropathy therapeutic agents. The available ALDRIs, like epalrestat and ranirestat, have certain drawbacks, such as toxicity and low solubility, prompting the need for safer and more effective inhibitors. This study used a structure‐based approach to search the PubChem database for new ALDR inhibitors with a Tanimoto coefficient greater than 0.8, with reference to epalrestat and ranirestat structures. The screened compounds demonstrated strong docking scores and favorable interactions with ALDR, particularly CID:45110135 and CID:58643777, which were further evaluated through ADMET and PASS analysis that provided insights into the pharmacokinetic and toxicity profiles of the compounds. In MD simulations, RMSD, RMSF, *R*g, SASA, hydrogen bond analysis, and PCA further analyzed the stability and dynamic characteristics of the ALDR‐ligand complexes. These simulations showed that the identified ligands preserved essential interactions that were observed during the docking process. The FEL analysis also provided additional evidence regarding the conformational stability of ALDR when bound to CID:45110135 and CID:58643777. However, this study does not include in vitro and in vivo validation, which should be addressed in future experimental studies. Taken together, this computational approach offers an understanding of ligand‐protein interactions, system behavior, and pharmacokinetic and toxicity properties. These outcomes provide a strong background for the development of novel therapeutic molecules for diabetic neuropathy.

## 4 Experimental Section

4.1

4.1.1

##### Computational Setup

The computational studies were conducted using an Intel workstation running Ubuntu 18.04.2 LTS. This setup allowed it to be compatible with other tools and processes necessary for computational drug discovery. A set of software applications and web resources was used to perform virtual screening, molecular docking, and MD simulations. InstaDock^[^ ^31^ ^]^ was used for docking and virtual screening analysis, as this tool has high‐throughput modes and quick affinity scoring. Molecular interaction analysis was done with the help of Discovery Studio Visualizer^[^ ^32^ ^]^ structural visualization was done using PyMOL^[^ ^33^ ^]^ and VMD^[^ ^34^ ^]^ for the trajectory of MD simulations. XMGRACE^[^ ^35^ ^]^ was employed to graph and analyze dynamic behavior and energy information of the protein‐ligand complexes. The sources used in the study also included PubChem for compound libraries, pkCSM^[^ ^36^ ^]^ for ADMET profiling, PASS^[^ ^19^ ^]^ for biological activity prediction, and the Protein Data Bank (PDB) for protein structure data.^[^ ^37^ ^]^ Altogether, these tools enabled a comprehensive approach to the integration and analysis of data in computational drug discovery

##### Preparation of the Target and Searching Epalrestat and Ranirestat‐Like Compounds

The 3D structure of ALDR was retrieved from the Protein Data Bank (PDB ID: 4LBS) as a high‐resolution model for computational analysis. The structure used in this study was prepared to enhance its conformation to be suitable for docking simulations. To clean the protein structure and refine the correct orientation of residues, the Swiss‐PDB Viewer^[^ ^38^ ^]^ was employed, while the MGL AutoDock Tools^[^ ^39^ ^]^ were used to translate the structure from PDB to PDBQT format required in InstaDock. Nonprotein groups were stripped off, and water molecules were omitted to show sites of interest, and then Kollman charges were added to hydrogen atoms, which were oriented at the polar state. The study aimed to identify structurally similar compounds to epalrestat and ranirestat for ligand preparation. A PubChem query was performed to retrieve compounds with ≥80% structural similarity, meeting Lipinski's rule of five to ensure favorable pharmacokinetics and drug‐like properties.^[^ ^40^ ^]^ The Tanimoto coefficient threshold of 0.8 was selected to ensure high structural similarity to epalrestat and ranirestat while maintaining sufficient diversity for novel inhibitor identification. This threshold balances pharmacological activity retention (via similarity) and exploration of chemical space for improved properties (via diversity).^[^ ^41^ ^]^ These compounds were subjected to energy minimization using the CHARMM force field in PyRx^[^ ^40^ ^]^ to achieve conformations with minimal steric clashes, enhancing their interaction potential during docking studies.

##### Virtual Screening and Molecular Docking

Virtual screening was performed to reduce the number of structurally similar compounds from a library to a reasonable number that could be subjected to molecular docking. Initial screening was performed based on the Lipinski rule of five, and every compound was docked against the ALDR through InstaDock. A blind docking was performed, and the software was able to search the entire protein for potential binding regions. The docking grid was established with the size of 66 Å × 55 Å × 88 Å in *x*, *y*, and *z* axes, with a grid point interval of 1 Å to accommodate the whole protein and its vicinity. Docking simulations calculated the binding affinity of each compound, which gave the strength of interaction between the protein and the ligand. The generated output files included binding conformations, interaction energies, and docking poses, which were used for screening.

##### Hit Selection and Drugability Assessment

After molecular docking, the first set of compounds was chosen according to their binding affinities, which were expressed as energy scores (Δ*G*). The set of compounds with essential interactions in the ALDR active site was chosen for the subsequent analysis. The selected compounds were subjected to physicochemical and pharmacokinetic analysis using pkCSM and CarcinoPred‐EL^[^ ^42^ ^]^ web tools. These tools could make ADMET predictions of absorption, distribution, metabolism, excretion, and toxicity parameters. In addition, the PASS analysis offered some information on the possible biological activities of the selected molecules. The docking and interaction outcomes were further supported by 2D and 3D molecular models of ALDR‐ligand complexes derived from Discovery Studio Visualizer. These visualizations stressed interaction residues and conformational stability, which supported the drug‐like nature of the selected compounds.

##### PASS Analysis

We utilized PASS analysis to explore chemical‐biological interactions and assess the potential biological activities of the compounds under investigation. The PASS server provides predictions in terms of Pa (probability of activity) and Pi (probability of inactivity) values, offering insights into the likelihood of a compound exhibiting specific biological properties.^[^ ^19^ ^]^ A higher Pa value indicates a greater likelihood of activity. Using this tool, we identified key biological attributes of the compounds, guiding the selection process for further studies. This approach allowed us to prioritize compounds with the highest potential for desired biological activity, contributing to a more efficient and targeted drug discovery strategy.

##### MD Simulations

MD simulations were performed on the selected compounds to validate docking results and evaluate the stability of the ALDR‐ligand complexes. Protein and ligand parameters were derived from the CHARMM36 force field.^[^ ^43^ ^]^ The simulation systems were solvated with a TIP3P water box using periodic boundary conditions with a 15 Å buffer surrounding the protein–ligand complex. To maintain system neutrality, Na^+^ and Cl^−^ ions were added to mimic physiological ionic strength. Energy minimization was performed to eliminate steric hindrance and to obtain a suitable starting structure. After that, the systems were heated from 0.1 to 300 K for 1 ns with a step of 3 K per picosecond. After this heating phase, a 100‐ps equilibration phase was applied to the system before the 500‐ns production run, which was performed using a 2‐fs integration time step. All simulations were performed at 300 K and 1 atm of pressure with the help of a Langevin thermostat and barostat.^[^ ^44^ ^]^ The long‐range electrostatics were computed using the Particle Mesh Ewald (PME) scheme,^[^ ^44^ ^]^ and short‐range interactions were cut off at 10 Å. The trajectory data points obtained from the simulation were also used to assess the structural dynamics, compactness, and time‐evolution analysis of the complexes to understand the structural stability of the complexes.

##### Essential Dynamics

Essential dynamics is a widely used computational technique to study the collective motions of a biomolecular system.^[^ ^30^ ^]^ PCA was performed to investigate the principal modes of motion and identify energetically favorable conformational states of the systems. This process involved the computation and diagonalization of the covariance matrix, which was calculated as follows

(1) $$C_{ij}=<\left(x_{i}-<x_{i}>\right)\left(x_{j}-<x_{j}>\right)>$$

where *x*_i_/*x*_j_ Is the coordinate of the *i*th/*j*th atom of the systems, and <–> represents an ensemble average.

FEL analysis is a widely used technique in computational biophysics and structural biology that provides critical insights into protein stability, folding pathways, and functional dynamics.^[^ ^30^ ^]^ By representing the energy landscape of a protein system in terms of collective variables, such as coordinates describing its conformational space, FEL analysis enables the identification of low‐energy states associated with stable protein conformations and the transition pathways between these states. This method offers a deeper understanding of the dynamic behavior and energetically favorable conformations of protein systems, shedding light on their stability and functional mechanisms. The FELs were constructed as follows

(2) $$\text{Δ}G\left(X\right)=-K_{B}T\ln\;P\left(X\right)$$

where *K*_B_ and *T* are the Boltzmann constant and absolute temperature, respectively. *P*(*X*) is the probability distribution of the molecular system along the PCs.

## Conflict of Interest

The authors declare no conflict of interest.

## Author Contributions

**Mohd Shahnawaz Khan**: conceptualization (equal); data curation (supporting); methodology (equal); validation (equal); visualization (equal); writing—original draft (equal). **Dharmendra Kumar Yadav**: data curation (equal); investigation (equal); visualization (equal); writing—review and editing (equal). **Moyad Shahwan**: data curation (equal); formal analysis (supporting); investigation (supporting); resources (supporting); software (supporting); writing—review and editing (equal); **Anas Shamsi**: conceptualization (lead); data curation (equal); formal analysis (supporting); funding acquisition (equal); investigation (equal); methodology (equal); supervision (lead); visualization (lead); writing—original draft (equal).

## Data Availability

All the data is presented in the manuscript.
